# Sacubitril/valsartan is well tolerated in patients with longstanding heart failure and history of cancer and improves ventricular function: real-world data

**DOI:** 10.1186/s40959-021-00121-y

**Published:** 2021-10-13

**Authors:** Maria Klara Frey, Henrike Arfsten, Noemi Pavo, Emilie Han, Stefan Kastl, Martin Hülsmann, Mariann Gyöngyösi, Jutta Bergler-Klein

**Affiliations:** Department of Cardiology, Medical University Vienna, Waehringer Guertel 18-20, 1090 Vienna, Austria

**Keywords:** Cardio-oncology, Heart failure, Cancer, Sacubitril-valsartan, Cardiotoxicity, ARNI, Natriuretic peptides

## Abstract

**Background:**

Sacubitril/valsartan has been shown to significantly reduce cardiovascular mortality and hospitalizations due to heart failure in patients with reduced ejection fraction (HFrEF) when compared to enalapril. Data about sacubitril/valsartan in patients with a history of cancer are scarce, as these patients were excluded from the pivotal trial, PARADIGM-HF. The aim of the current study was to assess tolerability of sacubitril/valsartan in patients with a history of cancer.

**Methods:**

We identified 225 patients at our heart failure out-patient unit who fulfilled the indication criteria to receive sacubitril/valsartan. Out of these, 9.3% (*n* = 21) had a history of histologically confirmed cancer. Oncologic surgery was performed in 16 (76.2%) patients, 11 (52.4%) patients received previous antineoplastic therapy and 9 patients (42.9%) radiation.

**Results:**

Sacubitril/valsartan was withdrawn in 3 of 21 patients (14.3%) because of dizziness (*n* = 2) or pruritus (*n* = 1). After a median follow-up of 12 months (range 1–34 months), NYHA functional class improved significantly from NYHA 3 to NYHA 2 (mean -0.6, *p* = 0.006) and left ventricular ejection fraction as assessed by echocardiography increased significantly from 26.8 ± 5.4% to 39.2 ± 10% (mean + 12%, CI 95% [8.4–16.4], *p* = 0.0004). NT-proBNP was significantly reduced (baseline median 2774 pg/ml, range 1441 – 12,982 vs follow-up 1266 pg/ml, range 199–6324, *p* = 0.009). There was no significant change in creatinine levels (1.18 ± 0.4 vs 1.22 ± 0.4 mg/dl; mean + 0.005 mg/dl, CI 95% [-0.21- 0.12], *p* = 0.566).

**Conclusions:**

In our pilot study we show that sacubitril/valsartan is generally well tolerated in patients with HFrEF and history of cancer. Importantly, even patients with long-standing cardiotoxicity induced heart failure can be treated and up-titrated with sacubitril/valsartan to usual target dosages, leading to improvement in LV function and biomarkers. Larger studies are needed to confirm these findings in cancer patients with cardiotoxicity.

## Background

The angiotensin receptor–neprilysin inhibitor sacubitril/valsartan has been shown to improve outcomes in patients with heart failure with reduced ejection fraction (HFrEF). In PARADIGM‐HF sacubitril/valsartan reduced the risk of cardiovascular death or heart failure (HF) hospitalization by 20% compared to enalapril [1]. International guidelines now recommend the introduction of sacubitril/valsartan in patients with HFrEF who remain symptomatic despite optimal uptitrated medical therapy [2].

Patients with a history of cancer were excluded in PARADIGM-HF as well as in following trials [3, 4]. Conventional chemotherapy is still among the most effective treatment options for many types of cancer. Cardiotoxicity leading to decrease in the left ventricular function impairs the prognosis of patients suffering from cancer. Recently, a large population-based observational study including more than 1 million patients with 28 different types of cancer, reported that 38% of patients died from cancer and 11.3% died from cardiovascular disease (CVD) [5]. Thus, CVD associated with cancer has become a major challenge for cardiologists and oncologists.

Until now, only few registries and small case reports have been published about sacubitril/valsartan in patients with cardiotoxicity induced cardiomyopathy [6–8]. The aim of the current study was to assess tolerability of sacubitril/valsartan in patients with a history of cancer in a real-world setting.

## Methods

The study was performed in accordance with the Declaration of Helsinki and approved by the local Ethics Committee of the Medical University of Vienna. Written informed consent was obtained from all study participants.

### Study population

This nonrandomized observational study is based on a prospective registry at the Medical University of Vienna, a university‐affiliated tertiary care center. Consecutive patients with stable chronic HFrEF in whom sacubitril/valsartan therapy was initiated were screened for history of cancer. The inclusion criteria were history of treated cancer with no current anticancer regimen during the present visit, left ventricular ejection fraction ≤ 35% and NYHA class ≥ 2 despite optimized guideline-directed medical therapy (GDMT), serum potassium ≤ 5.4 mmol/l, systolic arterial blood pressure ≥ 100 mmHg, estimated glomerular filtration rate (eGFR) ≥ 30 ml/min/1.73m^2^ based on central laboratory creatinine measurement and calculated using the Modification of Diet in Renal Disease formula.

### Baseline assessment and transthoracic echocardiography

Baseline parameter included clinical assessment defined by New York Heart Association (NYHA) class and routine blood sampling. Laboratory parameters including biomarkers such as serum N-terminal pro brain natriuretic peptide (NT-proBNP) were analyzed according to local laboratory standard procedures (Roche NT-proBNP Elecsys assay, Roche Diagnostics, Basel, Switzerland) as used in PARADIGM-HF [1]. Transthoracic echocardiography (TTE) was performed at baseline before initiation of sacubitril/valsartan and at follow-up visits by certified operators on high-end machines (GE Vivid E95 and Vivid 7; GE Healthcare, Wauwatosa, WI, USA) according to current recommendations [9]. Analysis was performed using an offline clinical workstation equipped with dedicated software (EchoPAC; GE Healthcare, Wauwatosa, WI, USA). The standard transthoracic echocardiography (TTE) protocol was extended by 2D speckle tracking analysis of left ventricular (LV) global longitudinal systolic strain (GLS) measured in an apical three-, four- and two-chamber view, as well as tissue doppler imaging (TDI) of the right ventricle (RV) [10].

### Statistical analysis

Data are expressed as mean ± standard deviation (SD) if normally distributed, or otherwise by median (interquartile range). Categorical variables are expressed as numbers and percentages. In all calculations, a *P*-value of < 0.05 was considered statistically significant and all analyses were performed using SPSS 22 (IBM Corp, NY, USA).

## Results

### Patient characteristics

In total, 21 patients out of 225 patients (9.3%) on sacubitril/valsartan had a history of histologically confirmed and treated cancer: 23.8% breast cancer (*n* = 5), 14.3% colorectal cancer (*n* = 3), 14.3% non-Hodgkin lymphoma (*n* = 3), 9.5% osteosarcoma (*n* = 2), 9.5% renal cell carcinoma (*n* = 2), lung cancer, Hodgkin lymphoma, prostate cancer, bladder carcinoma, pancreas carcinoma, multiple myeloma, acute leukaemia and myeoloproliferative syndrome (each 4.8%, *n* = 1). Surgery due to cancer was performed in 76.2% of patients (*n* = 16), 52.4% previously received antineoplastic therapy (*n* = 11) and. 42.9% radiation therapy (*n* = 9). Median time between start of cancer treatment and diagnosis of heart failure was 5.8 years (range 0.2–24.3 years), whereas median time between start of cancer treatment and start of sacubitril/valsartan was 9.7 years (range 0.5–38 years). GDMT was sufficiently up-titrated before start of sacubitril/valsartan in all patients: the recommended dose of beta-blockers and ACE inhibitors was achieved by 80% of patients respectively. Out of 21 patients with a history of cancer, 33.3% (*n* = 7) had previous coronary artery disease, the baseline characteristics and comorbidities are depicted in Table 1. An ICD (implantable cardioverter defibrillator) was present in 28.6% of patient, and a CRT (cardiac resynchronization therapy) device in 23.8% of patients.

**Table 1:** Baseline characteristics of the study population (*n* = 21)

Age (years)	70 (20–91)
Female sex, n (%)	10 (48)
BMI (mean ± SD)	25.1 ± 4.6
Cancer type	
Breast cancer, n (%)	5 (23.8)
Colorectal cancer, n (%)	3 (14.3)
Non-Hodgkin lymphom, n (%)	3 (14.3)
Osteosarcoma, n (%)	2 (9.5)
Renal cell carcinoma, n (%)	2 (9.5)
Lung cancer, n (%)	1 (4.8)
Hodgkin lymphom, n (%)	1 (4.8)
Bladder carcinoma, n (%)	1 (4.8)
Myeoloproliferative syndrome, n (%)	1 (4.8)
Prostate cancer, n (%)	1 (4.8)
Hypertension, n (%)	7 (33.3)
Hyperlipidemia, n (%)	5 (23.8)
Diabetes Mellitus, n (%)	5 (23.8)
Coronary Artery Disease, n (%)	7 (33.3)
Atrial Fibrillation/other arrhythmias, n (%)	9 (42.9)
ICD, n (%)	6 (28.6)
CRT, n (%)	5 (23.8)
Previous cancer treatment	
Antineoplastic agents	11 (52.4)
Thoracic radiation	9 (42.9)
Antineoplastic agents and radiation	6 (28.6)
Surgery	16 (76.2)
Previous use of medication	
ACE‐inhibitor or ARB, n (%)	21 (100)
Beta‐blocker, n (%)	21 (100)
Mineralocorticoid agonist, n (%)	19 (90.5)
Diuretic, n (%)	10 (47.6)
Digitalis, n (%)	1 (4.8)

Categorical variables expressed as frequencies (n) and percentages (%). Skewed variables presented as median (interquartile range)

*Abbreviations*: *BMI* Body mass index, *ICD* Implantable cardioverter defibrillator, *CRT* Cardiac resynchronization therapy, *ACE* Angiotensin converting enzyme, *ARB* Angiotensin receptor blocker

### Improvement of clinical status and laboratory parameters

**Fig. 1:**
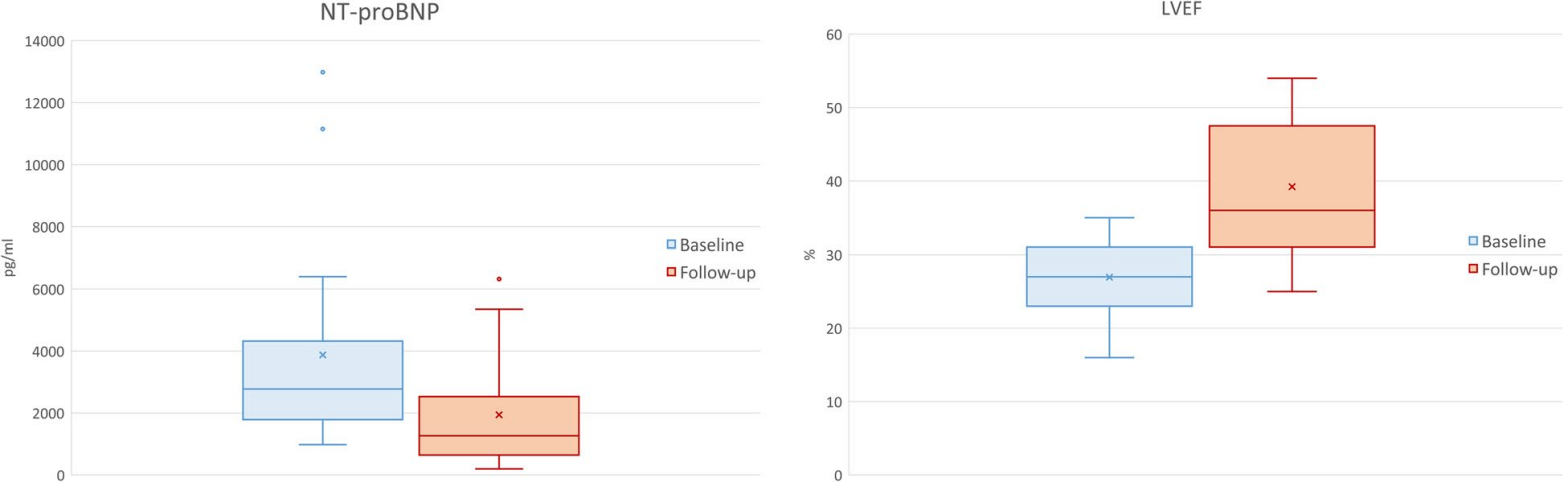
Decrease of NT-proBNP levels and improvement of LV-ejection fraction after treatment with sacubitril/valsartan (*n* = 21). *Abbreviations*: NT-proBNP N-terminal pro B-type natriuretic peptide, LV-EF left ventricular ejection fraction

Sacubitril/valsartan was well tolerated without significant side effects in 18 patients (85.7%). Sacubitril/valsartan was withdrawn in 3 patients (14.3%): two patients stopped medication because of dizziness after 16 and 7 months respectively, the third one because of pruritus 5 months after initiation. Two patients received only a medium dosage of sacubitril/valsartan 49/51 mg twice daily because of symptomatic hypotension, the remaining 16 patients were up-titrated to the high dosage of 97/103 mg twice daily. After a median follow-up of 12 months (range 2–34 months), NYHA functional class improved significantly (2.9 ± 0.4 vs 2.3 ± 0.6, *p* = 0.001) and NT-proBNP levels were significantly decreased (median 2774 pg/ml, range 1441 – 12,982 vs 1266 pg/ml, range 199–6324, *p* = 0.009) (Fig. 1) (Table 2). Importantly, there was no significant change in creatinine levels (1.18 ± 0.4 mg/dl vs 1.22 ± 0.4 mg/dl, *p* = 0.566) or serum potassium levels (4.52 ± 0.4 mmol/l vs 4.50 ± 0.4 mmol/l, *p* = 0.776 mmol/l). Systolic arterial blood pressure decreased significantly during follow-up (124 ± 15 vs115 ± 15 mmHg, *p* = 0.003), while heart rate did not differ between baseline and follow-up (72 ± 14 bpm vs 70 ± 11 bpm, *p* = 0.5) (Table 3). Interestingly, electrocardiogram (ECG) showed atrial fibrillation at baseline in 9 patients (42.9%) whereas only 5 patients (23.8%) had documented atrial fibrillation at follow-up. No other side effects were reported by the patients.

**Table 2:** Exploratory clinical and biomarker outcomes (*n* = 21)

	Baseline	Follow-up	*P*
NT-proBNP, pg/ml	3873 ± 3140	1945 ± 1860	0.009
NYHA class	2.9 ± 0.4	2.3 ± 0.6	0.001

*Abbreviations*: *NT-proBNP* N-terminal pro B-type natriuretic peptide, *NYHA* New York Heart Association

**Table 3:** Key safety outcomes (*n* = 21)

	Baseline	Follow-up	*P*
Creatinine, mg/dl	1.18 ± 0.4	1.22 ± 0.4	0.566
Serum potassium, mmol per liter	4.52 ± 0.4	4.50 ± 0.4	0.776
Systolic blood pressure, mmHg	124 ± 15	115 ± 15	0.003
Heart rate, bpm	72 ± 14	70 ± 11	0.494

### Improvement of echocardiographic parameters

Left ventricular ejection fraction as assessed by echocardiography increased strikingly (26.8% ± 5.4% vs 39.2% ± 10.0%, *p* = 0.0004) after 12 months (range 2–34 months) and also LV-GLS improved significantly (-8.1 ± 2.9% vs -12.4 ± 3.8%, *p* = 0.001) (Fig. 1). There was a tendency to reduction in left atrial volume after sacubitril/valsartan treatment (48.3 ± 19.6 vs 34.3 ± 23.1 ml/m^2^, *p* = 0.050), whereas LV end-diastolic volume decreased significantly (87.1 ± 31.7 vs 66.8 ± 37.2 ml/m^2^, *p* = 0.002) (Table 4). LV diastolic function was improved and regression of functional mitral regurgitation observed. Parameters of RV systolic function significantly improved as well (TAPSE 16.4 ± 2.6 vs 18.8 ± 2.6 mm, *p* = 0.0006) and RV tissue doppler imaging (TDI) (0.093 ± 0.02 versus 0.101 ± 0.02, *p* = 0.009). At baseline, 33.3% of patients (*n* = 7) had normal right ventricular function, whereas 42.9% (*n* = 9) showed normal RV function after treatment with sacubitril/valsartan.

**Table 4:** Echocardiographic parameters at baseline and follow-up (*n* = 21)

	Baseline	Follow-up	*P*
LA volume (ml/m^2^)	48.3 ± 19.6	34.3 ± 23.1	0.050
LVED volume (ml/ m^2^)	87.1 ± 31.7	66.8 ± 37.2	0.002
LV- EF (%)	26.8 ± 5.4	39.2 ± 10.0	0.0004
LV-GLS (-%)	8.1 ± 2.9	12.4 ± 3.8	0.001
Septal wall thickness (IVS, mm)	10.8 ± 1.8	10.9 ± 2.0	0.756
sPAP (mmHg)	46.5 ± 11.5	40.4 ± 18.2	0.156
RV TAPSE (mm)	16.4 ± 2.6	18.8 ± 2.6	0.0006
RV TDI (m/s)	0.093 ± 0.02	0.101 ± 0.02	0.009
Normal RV function, (n, %)	7 (33.3)	9 (42.9)	
LV diastolic function			
Grade I n (%)	1 (4.7)	5 (23.8)	
Grade II, n (%)	6 (28.6)	2 (9.5)	
Grade III, n (%)	5 (23.8)	-	
Tricuspid regurgitation (grade)	1.6 ± 0.8	1.4 ± 0.9	0.227
Mitral Rrgurgitation (grade)	2.0 ± 0.8	1.5 ± 0.5	0.008

*Abbreviations*: *LA* Left atrium, *LVED* Left ventricle end diastolic, *LV- EF* Left ventricular ejection fraction, *LV-GLS* Left ventricular global longitudinal strain, *LVF* Left ventricular function, *IVS* Intraventricular septum, *sPAP* Systolic pulmonary artery pressure, *TAPSE* Tricuspid anular plane systolic excursion, *RV TDI* Right ventricular tissue doppler imaging

## Discussion

In the present study we demonstrate that sacubitril/valsartan is efficient and well tolerated in patients with heart failure and a history of cancer. Only three patients out of 21 (14.3%) had to discontinue the medication because of mild side effects, e. g. dizziness. Importantly, no deterioration of renal function or hyperkalemia were observed.

In fact, the striking result of this study was an impressive rise in LV systolic function despite established optimal heart failure therapy in patients with even longstanding heart failure and history of cancer. Improvement in left ventricular ejection fraction was paralleled by a significant drop in natriuretic peptides. This observation is remarkable as it is believed that myocardial damage caused by cardiotoxic agents is related to “irreversible” necrosis of the cardiomyocytes [11]. Cardinale et al. showed that most commonly cardiotoxicity after anthracycline-containing therapy occurs within the first year and early treatment with angiotensin converting enzyme (ACE) inhibitors was crucial for a substantial recovery of cardiac function in this study [12]. Our results are in line with two recent publications, where LV ejection fraction improved after treatment with sacubitril/valsartan in patients treated with cardiotoxic cancer therapy [7, 8]. However, in our present study we show that not only left ventricular but also the right ventricular function can be improved with sacubitril/valsartan in cancer-treated patients, a finding which was not examined in the previous registries. Underlying mechanisms of this finding have to be elucidated, possibly the natriuretic effects of sacubitril can also relieve the right ventricular load.

Myocardial dysfunction and heart failure are the most predominant clinical presentations of cardiotoxicity associated with significant morbidity and mortality. Cardiotoxicity of anticancer therapy can vary substantially, occurring during/early after treatment or years later [13]. Cardiac damage can be transient or induce irreversible cell injury, as well as progressive myocardial fibrosis, e.g. after anthracyclines [14, 15]. Newer anticancer treatment such as immune checkpoint inhibitors, CAR-T treatment and other types of immunotherapy can induce myocarditis, arrhythmias, or myocardial ischemia leading to HF, as well as e.g. in tyrosine kinase inhibitors, cisplatin and other therapies, depending on baseline cardiac risk [15–17]. Thus, patients with a history of cancer do not always categorize as “classical cardiotoxicity-induced cardiomyopathy (CMP)”, but can present with heart failure due to arrhythmias, ischemia or after myocarditis. In addition, radiation therapy can cause interstitial myocardial fibrosis and may have synergistic effects on cardiac risk in combination with cardiotoxic chemotherapy [18, 19]. Apart from anticancer therapy, cancer itself has cardiotoxic effects independent of those caused by chemotherapy [20]. For instance, left ventricular mass is progressively lost and cardiac function becomes increasingly impaired in rodents with cancer cachexia [21]. In a prospective study enrolling more than 500 treatment naive cancer patients, increased resting heart rate was independently associated with all-cause mortality, especially in lung and gastrointestinal cancers [22]. There are several suggested common pathways like inflammation, stress, altered angiogenesis and genetic disposition potentially underlying both HF and cancer [23]. In the present study, the reduced LV ejection fraction after cancer therapy might have been enhanced by underlying cardiac comorbidities such as coronary artery disease (CAD) or hypertension present in a third of patients reflecting “real world”-cases often challenging physicians caring for these heterogenous group of patients [16]. We could observe an impressive rise in LV function despite previous optimal heart failure therapy when adding sacubitril/valsartan even in already longterm chronic heart failure due to cardiotoxic cancer treatment in a variety of cancer entities throughout different age groups. Therefore, treatment with sacubitril/valsartan appears to address many causes of heart failure induced by antineoplastic therapy, even at a late stage.

Another interesting finding of our study is that atrial fibrillation was less frequently present in patients after treatment with sacubitril/valsartan. This could be of special interest regarding patients with a history of cancer, as they are also at higher risk for for bleeding and/or thrombosis formation. Heart rhythm disorders are a common adverse effect of many antineoplastic treatments leading to high morbidity and hospitalisation rate in these patients. Reduced incidence of atrial fibrillation could be explained by a reduction in left atrial filling pressures, an important determinant of risk for AF recurrence [24]. This hypothesis was strengthened by the improvement in diastolic function at follow-up in our registry, and a tendency to reduction of left atrial volumes. Very recently it has been shown that sacubitril/valsartan attenuates atrial electrical and structural remodelling in a rabbit model of atrial fibrillation [25], which might also explain our findings.

Several limitations of this analysis should be noted. The small number of patients and the lack of a control group presents a limitation of the present study. Nevertheless, we assessed the tolerability of sacubitril/valsartan in this complex, vulnerable group of patients. When comparing our data with the hallmark study on sacubitril/valsartan, the PARADIGM-HF trial, baseline characteristics of our patients are quite comparable to patients without a history of cancer enrolled in PARADIGM-HF [1]. Also the significant drop in NT-pro BNP levels is comparable to data of this trial [26]. Whether an earlier start of sacubitril/valsartan might even enhance the ventricular recovery further, remains to be investigated.

## Conclusions

We were able to show that sacubitril-valsartan is well tolerated and significantly improves left and right ventricular function even in patients with longstanding chronic heart failure and a history of cancer. This pilot study might help to gain insights in treatment options for these patients with complex pathologies and multiorgan disease. Bearing in mind that cardiotoxicity of anticancer therapy is defined as a decline in LV EF of more than 10% to levels under 50% [11], sacubitril/valsartan initiation might be even more compulsory to prevent further myocardial damage and to enable the continuation of vitally important anticancer therapy. Further studies should address whether an immediate start at early signs of cardiotoxicity might hold back cardiac deterioration.

## Data Availability

The datasets used and/or analyzed during the current study are available from the corresponding authors on reasonable request.

## Abbreviations

ACE: Angiotensin converting enzyme
ARNI: Angiotensin receptor-neprilysin inhibitor
CI: Confidence interval
CMP: Cardiomyopathy
CRT: Cardiac resynchronization therapy
CVD: Cardiovascular disease
ECG: Electrocardiogram
eGFR: Estimated glomerular filtration rate
GDMT: Guideline-directed medical therapy
GLS: Global longitudinal systolic strain
HF: Heart failure
HFrEF: Heart failure with reduced ejection fraction
ICD: Implantable cardioverter defibrillator
LV: Left ventricle
NT-proBNP: N-terminal pro brain natriuretic peptide
NYHA: New York Heart Association
RV: Right ventricle
SD: Standard deviation
TDI: Tissue doppler imaging
TTE: Transthoracic echocardiography
